# Site-Resolved Plasma-Protein Architecture of Infection Susceptibility: A Cis-pQTL Mendelian Randomization and Colocalization Study

**DOI:** 10.3390/genes17080887

**Published:** 2026-07-29

**Authors:** Shan Ji, Tongzeng Li

**Affiliations:** Department of Infectious Diseases, Beijing You’an Hospital, Capital Medical University, Beijing 100071, China; jsshjdd@163.com

**Keywords:** plasma proteomics, pQTL, Mendelian randomization, colocalization, infection susceptibility, PSCA

## Abstract

**Background/Objectives:** Infection susceptibility and systemic infection severity arise from distinct tissue barriers and host-response compartments, but plasma-protein genetic studies often treat infection as a broad phenotype. We aimed to define a site-resolved host plasma-protein architecture of infection susceptibility across urinary, lower-respiratory, systemic, and skin/soft-tissue infection outcomes. **Methods:** We performed proteome-wide two-sample cis-protein quantitative trait locus (cis-pQTL) Mendelian randomization (MR) using UK Biobank Pharma Proteomics Project (UKB-PPP) instruments and registry-derived FinnGen R13 infection endpoints. Prioritized protein–outcome pairs underwent regional colocalization, prior-sensitivity analysis, Steiger-like directionality sensitivity, Finnish and broader European reference-panel linkage disequilibrium (LD) assessment, candidate-level deCODE pQTL-source follow-up where feasible, and druggability and safety-domain annotation. **Results:** Across 18,766 harmonized tests, evidence was strongly site-resolved. PSCA emerged as the principal novel cystitis-specific candidate (OR 0.977, 95% CI 0.967–0.987), with strong colocalization and high Finnish reference-panel linkage disequilibrium between the MR instrument and the top colocalization single-nucleotide polymorphism (SNP) (r^2^ = 0.980). UMOD anchored urinary host-defense coherence. APOE showed strong evidence for the registry-derived implicit sepsis endpoint but was interpreted as a pleiotropy-prone broad host-response or severity-associated locus. TNFSF8 retained a robust pneumonia MR signal, but colocalization was moderate (PP.H4 = 0.544) and became weak under stringent priors (PP.H4 = 0.107), despite high-LD deCODE proxy-instrument follow-up. Skin/soft-tissue infection lacked a convergent lead. **Conclusions:** This study supports a site-resolved architecture of genetically anchored plasma-protein candidates for infection susceptibility. PSCA and UMOD define complementary urinary signals, whereas APOE and TNFSF8 require cautious interpretation as broad host-response and lower-respiratory follow-up hypotheses rather than established therapeutic targets.

## 1. Introduction

Infection susceptibility and systemic infection severity are not a single biological entity. Pneumonia, urinary-tract infection, skin/soft-tissue infection, and sepsis originate at distinct epithelial barriers and only later converge through systemic inflammation, coagulation, and organ dysfunction [1,2]. This is why a generic infection phenotype can blur target interpretation: a urinary epithelial defense protein, a lung T-cell costimulatory ligand, and a pleiotropic lipoprotein pathway are unlikely to have the same clinical meaning across all infection sites.

Large-scale plasma-proteome resources now make genetically anchored protein prioritization feasible. Protein quantitative trait locus (pQTL) atlases from INTERVAL, deCODE, and UKB-PPP link inherited variation to circulating protein abundance and have been used to nominate disease mechanisms, drug-repurposing opportunities, and safety liabilities [3,4,5,6]. cis-pQTL instruments are especially useful because they anchor the exposure to the encoding locus, reducing but not eliminating concerns about horizontal pleiotropy. UKB-PPP was selected as the primary pQTL source because it provides one of the largest publicly available plasma-proteome genetic resources, with broad protein coverage, large sample size, and direct suitability for proteome-wide genetic target prioritization.

Previous proteomic and genetic studies have investigated acute sepsis signatures, mortality-associated proteomic endotypes, circulating immune proteins, and proteome-wide MR candidates for sepsis and respiratory-tract infections [2,7,8,9,10,11]. These studies demonstrate the value of proteomic and genetic approaches, but they have generally focused on a single broad endpoint, a specific infection, or a non-infectious immune phenotype. They have not directly compared a common plasma-protein instrument set across urinary, lower-respiratory, skin/soft-tissue, and systemic infection modules within one harmonized framework. The present study therefore extends previous work by asking whether genetically proxied protein associations and their regional architectures differ according to the anatomical and clinical site of infection.

Accordingly, the objective of this study was to define a site-resolved plasma-protein genetic architecture across major infection-susceptibility modules and to prioritize candidate proteins using convergent genetic and translational evidence. We compared urinary, lower-respiratory, skin/soft-tissue, and systemic infection outcomes and integrated cis-pQTL MR, regional colocalization, prior sensitivity, Steiger-like directionality sensitivity, reference-panel LD assessment, pQTL-source follow-up where feasible, druggability, and safety-domain evidence. The framework was designed for genetically anchored protein prioritization and hypothesis generation rather than definitive causal-mechanism assignment or estimation of pharmacological treatment effects.

## 2. Materials and Methods

### 2.1. Study Design and Data Sources

We conducted a two-sample, proteome-wide MR and protein-prioritization study. Plasma-protein instruments were derived from UKB-PPP cis-pQTL summary statistics, infection outcomes from FinnGen R13, and follow-up pQTL regions from ProteoNexus [5,12,13]. Candidate druggability and functional annotations were obtained from Open Targets and UniProt [14,15].

Primary infection outcomes represented four major infection-site modules: pneumonia, cystitis, skin/subcutaneous infection, and implicit sepsis. Sensitivity outcomes included bacterial pneumonia, lower-respiratory infection, pyelonephritis, cellulitis, cutaneous abscess, bacterial sepsis, and other septicemia to evaluate phenotype coherence within each module (Supplementary Materials, Table S1). The implicit sepsis phenotype was a registry-derived FinnGen endpoint rather than a clinically adjudicated sepsis endpoint. The cystitis and pyelonephritis outcomes were combined-sex registry-derived endpoints; sex-specific case counts were not available in the public summary metadata. Overall analysis dimensions are summarized in Supplementary Materials, Table S2.

### 2.2. pQTL Instruments and Mendelian Randomization

We used cis-pQTL instruments from UKB-PPP summary statistics and excluded variants in the major histocompatibility complex (MHC) region to reduce HLA-driven pleiotropy. The source instrument table contained 1952 allele-specific cis-pQTL rows, corresponding to 1951 unique protein–rsID instrument pairs across 1950 proteins. The one-row difference reflected two distinct allele-specific records for the multiallelic GPHA2 variant rs200683903 rather than an exact duplicate. After allele harmonization and requiring outcome-variant availability across FinnGen outcomes, 1706 proteins were retained in the harmonized outcome-comparable testing universe, yielding 18,766 protein–outcome MR tests across 11 outcomes.

Among the 1950 proteins, 1949 (99.95%) were instrumented by a single rsID and therefore required Wald-ratio estimation; only one protein had two eligible rsID instruments and was analyzed using inverse-variance weighting. F statistics were available for all 1951 unique protein–rsID instruments. The median F statistic was 941.81 (interquartile range, 250.87–3529.62; range, 45.37–37,472.46), and no instrument had an F statistic below 10. The estimates were interpreted as genetically anchored protein-prioritization signals rather than precise causal or pharmacological effect estimates [16]. Instrument-level and candidate-level strength summaries are provided in Supplementary Materials, Tables S2–S4.

We applied false-discovery-rate (FDR) correction across all tested protein–outcome pairs and separately within the four primary outcomes. Proteins were advanced to detailed evaluation when they showed primary-outcome FDR support, coherent sensitivity-outcome evidence, or a biologically interpretable site-specific MR signal warranting regional assessment. Advancement to colocalization did not itself designate a protein as a final lead.

### 2.3. Regional Colocalization and Prior Sensitivity

For prioritized candidates, we extracted ±1 Mb pQTL regions from ProteoNexus and matched them to FinnGen outcome loci by rsID. We used coloc.abf to estimate posterior probabilities for five hypotheses, with PP.H4 representing support for a shared regional causal variant [17]. For descriptive interpretation, PP.H4 ≥ 0.80 was labeled strong, PP.H4 of 0.50–0.80 moderate, and PP.H4 of 0.20–0.50 weak or suggestive. These categories were pragmatic labels rather than absolute decision boundaries because posterior probabilities depend on prior assumptions, variant coverage, regional LD, and the possibility of multiple causal signals [18,19]. Accordingly, moderate or prior-sensitive PP.H4 values were not treated as definitive evidence of a shared causal variant.

To evaluate robustness to prior assumptions, we repeated colocalization under four settings: default, conservative shared-causal prior, stringent shared-causal prior, and a low-p1/p2 stringent model [18]. Signals that remained moderate or strong across settings were considered more prior-robust than signals that declined markedly under stricter assumptions. Regional colocalization and prior-sensitivity outputs are summarized in Supplementary Materials, Tables S5 and S6. Because the top ProteoNexus/FinnGen colocalization SNP was not always identical to the UKB-PPP MR instrument, physical distance was retained only as a descriptive quality-control metric and was not interpreted as evidence of LD or a shared causal variant.

### 2.4. Directionality Sensitivity and Reference-Panel Linkage Disequilibrium Assessment

For manuscript-priority protein–outcome pairs, we performed a Steiger-like directionality sensitivity analysis using the validated merged pQTL–FinnGen regional SNP set used for colocalization. Approximate variance explained was estimated from pQTL and outcome z statistics, and a pair was considered directionally supported when most regional variants showed greater approximate variance explained for the protein than for the infection outcome. Because circulating protein abundance and binary infection outcomes differ in scale, ascertainment, and measurement assumptions, this analysis was interpreted as sensitivity evidence rather than definitive proof of causal direction.

We additionally estimated pairwise LD between the UKB-PPP MR instrument and the top colocalization SNP and, where applicable, between the UKB-PPP and deCODE instruments. LD was calculated using LDlinkR with the high-coverage 1000 Genomes Finnish (FIN) population as the primary reference and the broader European (EUR) population as a sensitivity reference [20]. Pairwise r^2^ was used as the principal measure of variant correlation. For descriptive reporting, r^2^ ≥ 0.80 was labeled high LD, 0.20 ≤ r^2^ < 0.80 intermediate LD, and r^2^ < 0.20 low LD. These reference-panel estimates were used to distinguish likely proxy instruments from regional follow-up signals; they were not interpreted as individual-level FinnGen LD or proof of a shared causal variant. Directionality, physical-distance, and LD outputs are summarized in Supplementary Materials, Table S12.

### 2.5. Candidate-Level pQTL-Source Follow-Up

To evaluate whether prioritized associations depended on the UKB-PPP instrument source, we performed candidate-level pQTL-source follow-up using deCODE plasma-proteome pQTL summary statistics where suitable instruments were available. Genome-wide significant regional cis-pQTLs (*p* < 5 × 10^−8^ and F > 10) were selected and tested against the corresponding FinnGen outcomes using Wald-ratio estimates. Pairwise FIN and EUR LD between the UKB-PPP and deCODE instruments was examined to determine whether the external analysis represented high-LD proxy-instrument follow-up or a distinct regional pQTL-source analysis. These analyses were not treated as universal external validation.

### 2.6. Druggability Annotation and Safety-Domain Scanning

We annotated candidate proteins using Open Targets and UniProt to assess target identity, tractability evidence, approved or clinical modality signals, subcellular localization, and functional context [14,15]. We also performed a broad PheWAS-like safety-domain scan across cardiovascular, metabolic, renal, immune, hematologic, neurologic, respiratory, cancer, and other domains. This step was used to identify liabilities that could alter translational priority.

For final interpretation, candidates were classified using a tiered evidence framework. Tier 1 candidates showed FDR-supported MR evidence together with strong or prior-robust regional colocalization and coherent infection-site biology. Tier 2 candidates showed robust MR evidence but moderate, prior-sensitive, or otherwise unresolved regional architecture and were retained as follow-up hypotheses. Tier 3 candidates had MR evidence but weak colocalization, low local LD, broad safety liabilities, or limited site specificity and were retained as cautionary comparators. TGFB1 was evaluated in detail because it provided a tractability-positive but genetically evidence-limited comparison, whereas NUDT15 was retained as a supplementary MR-only signal. These tiers were interpretive categories rather than additional statistical significance thresholds.

### 2.7. Software and Computational Environment

All analyses and data processing were performed in R version 4.6.0 (2026-04-24 ucrt). Regional colocalization analyses were conducted using the coloc package (version 5.2.3), and reference-panel linkage disequilibrium analyses were performed using the LDlinkR package (version 1.4.0). Other analyses, including MR estimation, multiple-testing correction, and downstream data processing, were implemented in R using study-specific scripts.

## 3. Results

### 3.1. Proteome-Wide Screening Across Site-Specific Infection Outcomes

The source cis-pQTL table contained 1952 allele-specific rows, corresponding to 1951 unique protein–rsID instrument pairs across 1950 proteins. The one-row difference reflected two allele-specific records for the multiallelic GPHA2 variant rs200683903. Of the 1950 proteins, 1949 (99.95%) had a single rsID instrument and therefore required Wald-ratio estimation. F statistics were available for all 1951 unique protein–rsID instruments, with a median of 941.81 (interquartile range, 250.87–3529.62; range, 45.37–37,472.46); no instrument had F < 10.

**Figure 1:**
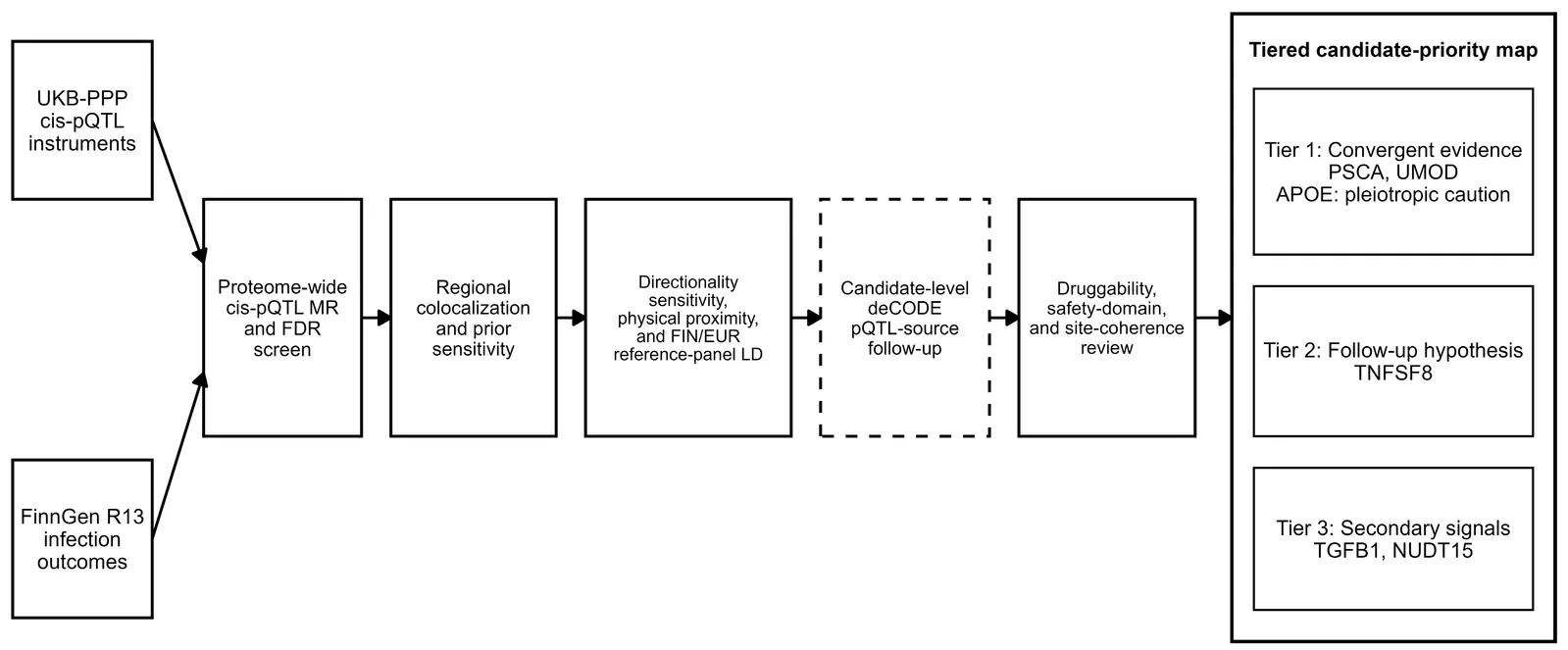
Study workflow and tiered candidate-prioritization framework. Solid boxes indicate the core analysis steps applied in the main workflow, whereas the dashed box indicates candidate-level deCODE pQTL-source follow-up performed only when a suitable external cis-pQTL instrument was available. Solid arrows denote steps applied across the planned analysis workflow. The dashed deCODE step denotes candidate-level pQTL-source follow-up performed only when a suitable external cis-pQTL instrument was available; it was not used as a universal validation requirement. The final panel visualizes the interpretive tiers: Tier 1 candidates combined FDR-supported MR evidence with strong or prior-robust colocalization and coherent infection-site biology; Tier 2 candidates had robust MR evidence but unresolved regional architecture; and Tier 3 or supplementary candidates were limited by weak colocalization, low LD, safety liabilities, or limited site specificity.

After harmonization, the analysis included 1706 proteins, 11 FinnGen infection outcomes, four primary infection-site outcomes, seven sensitivity outcomes, and 18,766 protein–outcome MR tests (Table 1; Figure 1; Supplementary Materials, Tables S1–S3). The workflow integrated proteome-wide cis-pQTL MR, regional colocalization, prior-sensitivity analysis, Steiger-like directionality sensitivity, descriptive physical-proximity assessment, FIN/EUR reference-panel LD evaluation, candidate-level pQTL-source follow-up, druggability annotation, and safety-domain scanning.

**Figure 2:**
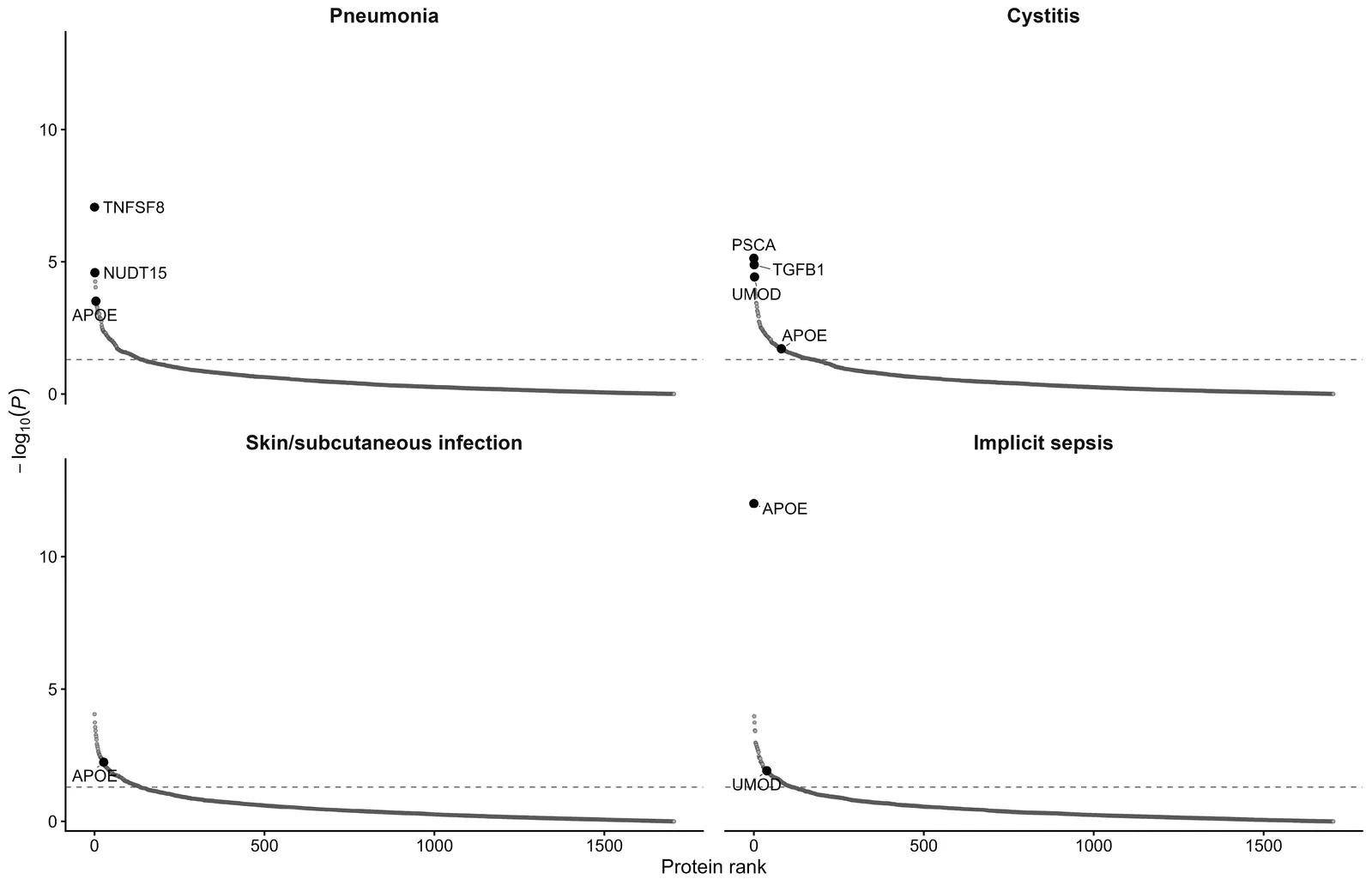
Proteome-wide Mendelian randomization results for the four primary registry-derived FinnGen infection endpoints. Each panel shows one primary infection outcome. Proteins are ranked on the x-axis according to their MR *p* value, with the strongest association shown at the left. The y-axis shows −log10(p). Points represent individual protein–outcome MR tests. The solid curve connects the ranked −log10(p) values to show the overall distribution of association strength across the proteome. The dashed horizontal line marks nominal *p* = 0.05. Selected candidate proteins are labeled to orient the reader; labels do not by themselves denote successful colocalization or therapeutic prioritization.

At the primary-outcome level, PSCA and UMOD defined the strongest urinary module, APOE showed the strongest MR association with the registry-derived implicit sepsis endpoint, and TNFSF8 was the leading pneumonia MR follow-up signal. No protein combined FDR-supported MR evidence with supportive regional colocalization for skin/subcutaneous infection (Figure 2; Table 2; Supplementary Materials, Table S4).

Integrated prioritization across MR evidence, regional colocalization, prior sensitivity, directionality sensitivity, reference-panel LD, biological coherence, druggability, and safety annotation identified PSCA as the principal novel cystitis-specific candidate and UMOD as the urinary-tract biological anchor. APOE was retained as a pleiotropy-prone broad host-response or severity-associated locus, whereas TNFSF8 was retained as a lower-respiratory follow-up hypothesis with unresolved shared-causal architecture (Table 2).

### 3.2. Urinary-Tract Infection Module: PSCA as a Cystitis-Specific Signal and UMOD as a Urinary Anchor

The urinary-tract module showed the most coherent site-resolved evidence. Genetically predicted higher PSCA was associated with lower cystitis risk (OR 0.977, 95% CI 0.967–0.987; *p* = 7.44 × 10^−6^; primary FDR = 0.0169), with strong regional colocalization (PP.H4 = 0.954) and moderate-to-strong support under stringent priors (PP.H4 = 0.676). Directionality sensitivity was supportive. The UKB-PPP MR instrument and top colocalization SNP were in high LD in FIN (r^2^ = 0.980) and EUR (r^2^ = 0.941), indicating that the MR and colocalization analyses captured highly correlated local variation. PSCA showed weaker evidence for pyelonephritis and other infection modules, supporting a cystitis-specific rather than broad urinary or systemic interpretation.

UMOD was retained as the urinary-tract biological anchor. Genetically predicted higher UMOD was associated with lower pyelonephritis risk (OR 0.952, 95% CI 0.934–0.971; *p* = 6.47 × 10^−7^), with additional support for cystitis. Regional colocalization was strong for pyelonephritis (PP.H4 = 0.995) and cystitis (PP.H4 = 0.910), remained robust under stringent priors, and showed supportive directionality. The MR and top colocalization SNPs were in high LD in FIN (r^2^ = 0.947) and EUR (r^2^ = 0.982). Thus, UMOD provides a positive biological anchor for urinary host-defense coherence, whereas PSCA represents the more cystitis-specific novelty signal.

### 3.3. Pneumonia and Lower-Respiratory Infection Module: TNFSF8 Follow-Up and APOE Broad Host-Response Signals

Genetically predicted higher TNFSF8 was associated with increased pneumonia risk (OR 1.142, 95% CI 1.088–1.199; *p* = 8.54 × 10^−8^; primary FDR = 2.91 × 10^−4^). However, regional colocalization was only moderate (PP.H4 = 0.544), with PP.H3 = 0.456, and became weak under the stringent prior (PP.H4 = 0.107). The UKB-PPP MR instrument and top colocalization SNP were in high LD in FIN (r^2^ = 0.979) and EUR (r^2^ = 0.992). This high LD establishes local variant correlation but does not resolve whether the same causal signal underlies protein abundance and pneumonia susceptibility. TNFSF8 was therefore retained as a lower-respiratory follow-up hypothesis rather than a near-definitive pneumonia target.

APOE showed strong regional colocalization with lower-respiratory infection (PP.H4 approximately 1.000) and moderate support for pneumonia (PP.H4 = 0.756). The UKB-PPP APOE MR instrument and top colocalization SNP showed intermediate LD in FIN (r^2^ = 0.539) and EUR (r^2^ = 0.669). Together with the extensive pleiotropy of the APOE locus, these results support interpretation as a broad host-response or severity-associated signal rather than a lung-specific susceptibility mechanism or direct therapeutic target.

Candidate-level deCODE pQTL-source follow-up further clarified the instrument relationships (Supplementary Materials, Table S11). The deCODE and UKB-PPP TNFSF8 instruments were in high LD in FIN (r^2^ = 0.957) and EUR (r^2^ = 0.910). The deCODE instrument showed directionally concordant associations with pneumonia (OR 1.229, 95% CI 1.135–1.331; *p* < 1 × 10^−4^), bacterial pneumonia (OR 1.243, 95% CI 1.078–1.432; *p* = 0.00268), and lower-respiratory infection (OR 1.138, 95% CI 1.006–1.287; *p* = 0.03924). This represents high-LD proxy-instrument follow-up but does not overcome the moderate and prior-sensitive colocalization result.

In contrast, the deCODE APOE instrument was in low LD with the UKB-PPP instrument in FIN (r^2^ = 0.084) and EUR (r^2^ = 0.060). Its borderline implicit-sepsis estimate was directionally aligned with the primary APOE result (OR 0.946, 95% CI 0.893–1.003; *p* = 0.06222), but lower-respiratory estimates were nonuniform. The deCODE APOE analysis therefore represents regional pQTL-source follow-up rather than proxy-instrument replication.

### 3.4. Implicit Sepsis and Systemic Host Response: APOE as a Pleiotropy-Prone Locus

**Figure 3:**
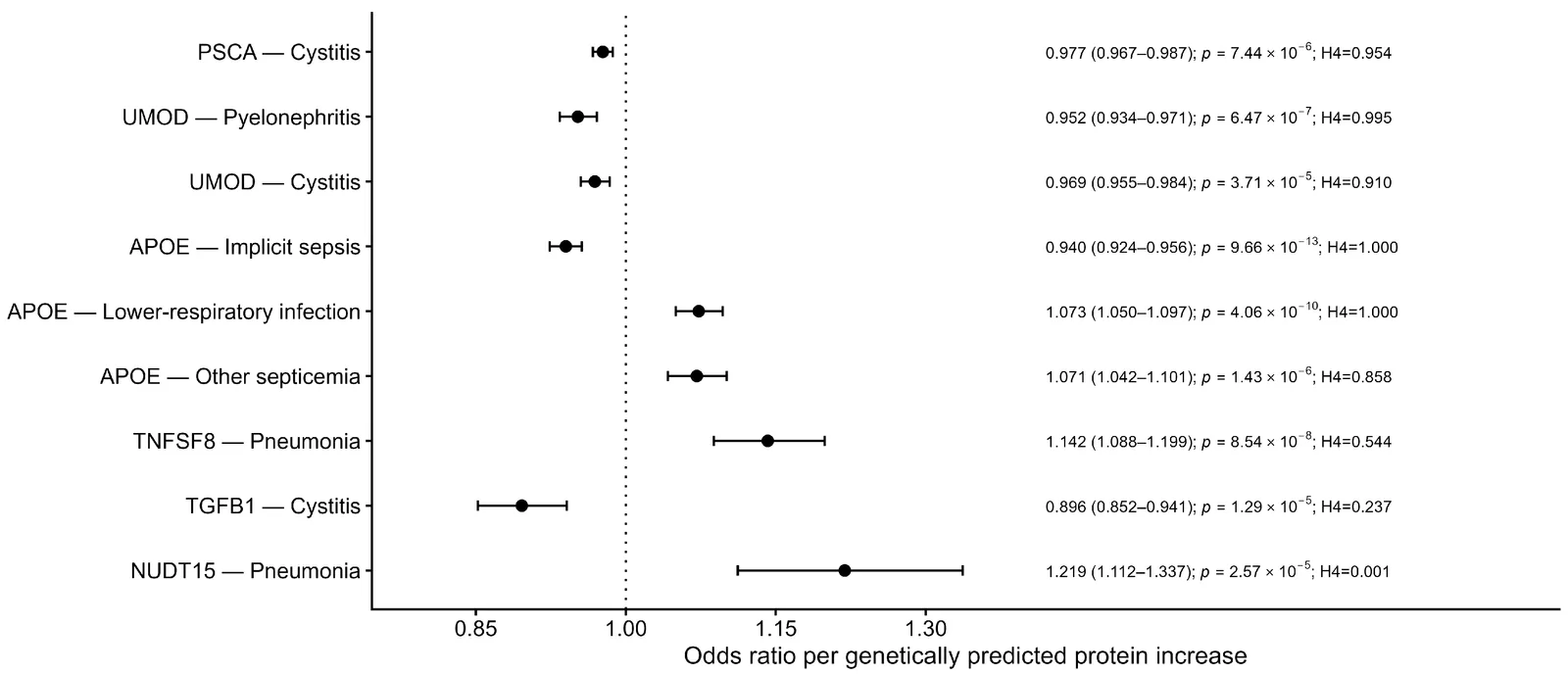
Mendelian randomization estimates and regional colocalization support for selected protein–outcome pairs. Points and horizontal lines show odds ratios and 95% confidence intervals per genetically predicted increase in protein abundance. The accompanying text reports MR *p* values and PP.H4. PP.H4 reflects regional sharing under the prespecified colocalization model and should be interpreted together with prior-sensitivity, directionality and LD analyses.

APOE showed the strongest primary MR association with the registry-derived implicit sepsis endpoint (OR 0.940, 95% CI 0.924–0.956; *p* = 9.66 × 10^−13^) and strong colocalization for implicit sepsis (PP.H4 = 0.9998) and other septicemia (PP.H4 = 0.858). Prior-sensitivity analysis supported robust strong or moderate-to-strong regional evidence. Nevertheless, because APOE is a highly pleiotropic lipid, cardiovascular, neurologic, immune, and inflammatory locus, the observed associations were interpreted as genetically anchored broad host-response or severity-related evidence rather than infection-specific causality or direct target validation. Selected MR estimates and corresponding regional colocalization support for prioritized protein–outcome pairs are summarized in Figure 3.

### 3.5. Skin/Soft-Tissue Infection and Secondary Candidates

For skin/subcutaneous infection, no candidate combined FDR-level MR evidence with supportive regional colocalization. This negative module is informative: it indicates that the screen did not simply identify generic inflammatory proteins across all infection phenotypes, and it supports the site-specific architecture of the final protein-priority map.

TGFB1 was associated with cystitis and showed favorable tractability annotation, but regional colocalization was weak (PP.H4 = 0.237), declined under stringent priors (PP.H4 = 0.030), and the MR and top colocalization SNPs were in low LD in FIN (r^2^ = 0.049) and EUR (r^2^ = 0.042). Its safety-domain annotation was also broad. NUDT15 showed a pneumonia MR association but no meaningful colocalization (PP.H4 = 0.001) and low MR–colocalization SNP LD in FIN (r^2^ = 0.053) and EUR (r^2^ = 0.010). TGFB1 was therefore retained as a tractability-positive but evidence-limited cautionary candidate, whereas NUDT15 was retained only as a supplementary MR-only signal.

### 3.6. Integrated Site-Specific Protein-Priority Map

**Figure 4:**
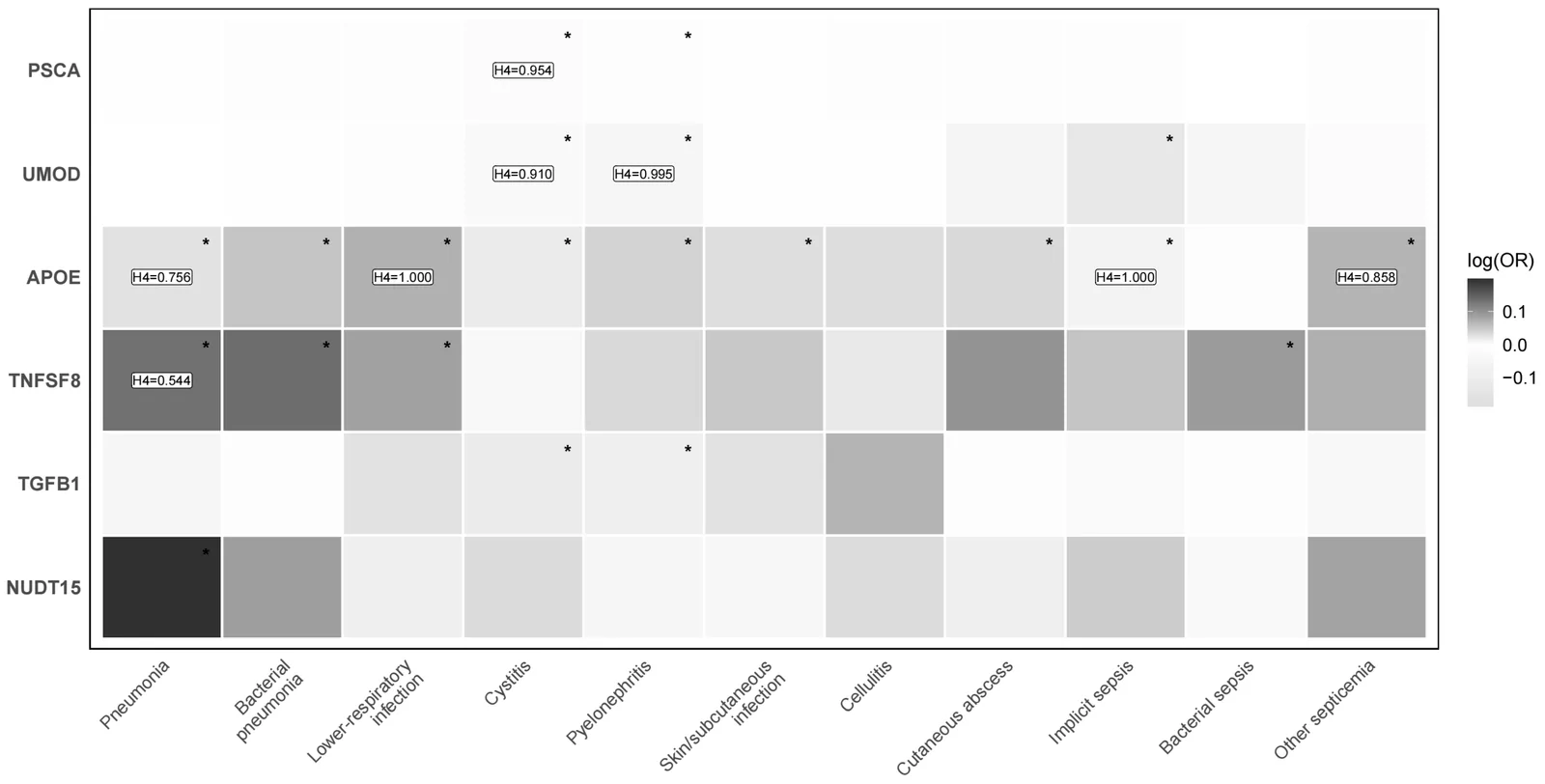
Site-resolved MR–colocalization matrix for prioritized proteins across 11 FinnGen infection outcomes. Cell shading represents log odds ratios from MR; asterisks (*) denote nominal MR *p* < 0.05. Boxed H4 labels report PP.H4 values to three decimal places when PP.H4 ≥ 0.50. The matrix is descriptive: statistical significance, colocalization, and therapeutic actionability are separate evidence dimensions, and unlabeled cells should not be interpreted as proof of no biological relationship.

The integrated protein-priority map separated candidates into a PSCA-first cystitis module, a UMOD urinary-anchor module, an APOE broad host-response/severity module, and a TNFSF8 lower-respiratory follow-up module (Table 3; Figure 4; Supplementary Materials, Tables S7, S8 and S12). PSCA and UMOD combined MR, strong colocalization, prior robustness, supportive directionality, and high FIN LD. APOE combined strong MR and colocalization with substantial pleiotropy and only intermediate MR–colocalization SNP LD. TNFSF8 showed robust MR and high-LD external follow-up but remained limited by moderate and prior-sensitive colocalization. TGFB1 and NUDT15 remained secondary or cautionary signals.

## 4. Discussion

This proteome-wide cis-pQTL MR and colocalization study supports a site-resolved architecture of genetically anchored plasma-protein candidates for infection susceptibility. Its main contribution is not a generic list of inflammatory markers, but an evidence-triage framework that distinguishes cystitis-specific epithelial susceptibility, broader urinary defense, lower-respiratory follow-up biology, systemic host-response or severity signals, and an informative negative skin/soft-tissue module. The additional instrument-strength and reference-panel LD analyses improved interpretability by distinguishing strong instruments and likely proxy variants from merely proximal regional signals.

More broadly, several statistically significant estimates in the prioritized set were modest in magnitude. These estimates represent lifelong genetically proxied differences in circulating protein abundance and should not be interpreted as the expected magnitude of pharmacological modulation. Their principal value lies in identifying biologically coherent pathways and prioritizing hypotheses for further investigation rather than directly predicting clinical treatment effects.

The urinary-tract findings were the most coherent and separated into two complementary signals. PSCA was the more novel cystitis-specific candidate. Its estimated association was modest (OR 0.977), and this magnitude should not be interpreted as the expected effect of pharmacological modulation. A cis-pQTL MR estimate reflects lifelong genetically proxied differences in circulating protein abundance, whereas a therapeutic intervention may differ in timing, magnitude, tissue distribution, and direction. For a common condition such as cystitis, a small genetically mediated effect may nevertheless identify a biologically relevant epithelial pathway. High FIN LD between the PSCA MR instrument and top colocalization SNP strengthened local coherence but did not establish a molecular mechanism. Proposed links to epithelial surface organization, bacterial adhesion niches, urothelial renewal, and injury–repair responses remain hypotheses that were not directly evaluated in this study. Given PSCA’s urothelial and oncologic context and the safety-domain findings (Supplementary Materials, Table S9), it is best viewed as a mechanistic susceptibility probe and prioritization candidate rather than an established intervention target.

UMOD, in contrast, is kidney-derived, abundant in urine, and positioned at the epithelial–luminal interface where ascending urinary infection begins [21,22]. Its cystitis/pyelonephritis MR coherence, strong and prior-robust colocalization, supportive directionality, and high FIN LD between the MR and top colocalization SNPs reinforce its role as the biological anchor of the urinary module. Existing MR evidence for upper-urinary-tract infection further supports this interpretation [23]. Together, PSCA and UMOD suggest complementary lower-urothelial and renal/urinary host-defense components, although neither signal alone establishes the direction or safety of therapeutic modulation.

APOE was statistically compelling but mechanistically and translationally complex. Sepsis proteomic studies have identified coordinated immune, complement, coagulation, metabolic, and tissue-injury states [2,7,9], and experimental studies support relationships between APOE genotype, apoE biology, and septic outcomes [24,25]. Nevertheless, APOE is among the most pleiotropic loci in the human genome. Its associations may reflect broad lipid, vascular, inflammatory, frailty, or host-response pathways rather than infection-specific susceptibility. The intermediate LD between the UKB-PPP instrument and top colocalization SNP and the low LD between the UKB-PPP and deCODE instruments further argue against describing the external analysis as direct replication. APOE should therefore be retained as a pleiotropy-prone broad host-response or severity-associated locus for mechanistic and safety interpretation, not as a straightforward infection target.

TNFSF8 illustrates why robust MR evidence, high-LD proxy follow-up, and biological plausibility are insufficient to establish a shared-causal or therapeutic signal when regional colocalization remains unresolved. CD30/CD30L participates in the TNF/TNFR costimulatory network and may influence T-cell activation and tissue-local immune memory [26,27,28,29]. These pathways provide a plausible context for lower-airway host responses, but the specific proposed mechanisms were not tested here. The UKB-PPP instrument was in high LD with both the top colocalization SNP and the deCODE instrument, supporting interpretation of the external analysis as proxy-instrument follow-up. However, PP.H4 was only 0.544, PP.H3 was 0.456, and stringent-prior PP.H4 declined to 0.107. TNFSF8 therefore remains a lower-respiratory follow-up hypothesis requiring ancestry-matched fine-mapping and experimental validation, rather than a near-definitive pneumonia target.

The druggability and safety layers materially changed candidate interpretation. TGFB1 had stronger conventional tractability but weak, prior-sensitive colocalization, low LD between the MR and top colocalization SNPs, and broad immune, fibrotic, oncologic, cardiovascular, and respiratory safety concerns, consistent with the pleiotropy of TGF-β signaling [30,31]. Conversely, UMOD had weaker conventional modality evidence but substantially stronger site coherence. These contrasts reinforce that tractability alone is insufficient for prioritization and that genetic, regional, tissue, and safety evidence should be considered jointly. Candidate-level safety-domain findings and the corresponding interpretive safeguards are summarized in Supplementary Materials, Tables S9 and S10.

A practical translational roadmap should therefore proceed in stages. First, prioritized loci require ancestry-matched fine-mapping and replication in diverse pQTL and infection GWAS resources. Second, circulating-protein signals should be connected to tissue- and cell-specific expression, particularly urothelial, renal tubular, pulmonary epithelial, and resident immune compartments [32]. Third, perturbation studies should determine whether increasing or decreasing the candidate protein alters bacterial adhesion, epithelial injury, immune-cell activation, or infection severity in relevant cellular and animal models. Finally, tissue specificity, direction of modulation, oncology risk, immune effects, and other safety liabilities must be evaluated before any candidate is considered therapeutically actionable.

The absence of a robust skin/soft-tissue lead is also informative. Rather than weakening the analysis, this finding supports the conclusion that host plasma-protein architecture differs across infection sites. It also reduces the risk that the study is merely capturing generic inflammation, because a generic inflammatory signature would be expected to appear across all major infection outcomes.

Several limitations should be considered. First, 1949 of 1950 proteins (99.95%) were instrumented by a single cis-pQTL and therefore relied on Wald-ratio estimation. Although all unique instruments were strong (minimum F = 45.37), single-instrument analyses cannot support conventional multi-instrument diagnostics such as MR-Egger regression, weighted-median estimation, or between-instrument heterogeneity testing. The estimates remain vulnerable to locus-specific horizontal pleiotropy, local LD structure, pQTL measurement error, and violations of the exclusion-restriction assumption. They should therefore be interpreted as genetically anchored protein-prioritization signals rather than precise causal or pharmacological effect estimates.

Second, colocalization depends on prior assumptions, variant coverage, LD structure, and the single-causal-variant approximation; moderate or prior-sensitive signals remain uncertain. Reference-panel LD improved interpretation of local SNP relationships, but FIN and EUR estimates from 1000 Genomes are not individual-level FinnGen LD and do not prove a shared causal variant. The Steiger-like directionality analysis was z-score-based and should also be viewed as sensitivity evidence. FinnGen is a Finnish founder population with distinctive allele frequencies and LD patterns, whereas UKB-PPP and the other primary pQTL resources are predominantly European ancestry. Portability to Asian, African, admixed, and other populations therefore requires validation. The urinary outcomes were combined-sex endpoints, and sex-specific case counts were unavailable in the public summary metadata; because cystitis and pyelonephritis are female-predominant conditions, the observed signals may disproportionately reflect female susceptibility biology and cannot be assumed to generalize equally to both sexes.

Third, the implicit sepsis endpoint was registry-derived rather than clinically adjudicated. Candidate-level deCODE follow-up was feasible for only a subset of proteins, and the APOE analysis represented regional rather than proxy-instrument follow-up. Residual participant overlap between resources cannot be completely excluded. Plasma abundance may not represent tissue-local protein activity at epithelial surfaces or immune synapses, and proposed biological mechanisms were not directly tested. Finally, druggability and safety-domain annotations were screening tools rather than evidence of clinical efficacy or acceptable therapeutic safety.

Despite these limitations, the study provides a focused protein-priority map for site-specific infection susceptibility. PSCA and UMOD formed the strongest urinary module, APOE marked a pleiotropy-prone broad host-response or severity signal, and TNFSF8 warranted cautious lower-respiratory follow-up. The value of the framework lies in distinguishing convergent evidence from MR-only, prior-sensitive, pleiotropic, or safety-limited signals rather than converting statistical associations directly into therapeutic claims.

## 5. Conclusions

This proteome-wide genetic study supports a site-resolved architecture of genetically anchored plasma-protein candidates for infection susceptibility. PSCA emerged as the principal novel cystitis-specific susceptibility candidate and mechanistic probe, whereas UMOD anchored broader urinary host-defense coherence. APOE represented a strong but pleiotropy-prone broad host-response or severity-associated locus, and TNFSF8 remained a lower-respiratory follow-up hypothesis with unresolved shared-causal architecture. These findings do not establish definitive therapeutic targets, but they demonstrate that integrating MR, regional colocalization, prior sensitivity, directionality sensitivity, reference-panel LD, infection-site coherence, druggability, and safety provides a more transparent prioritization framework than MR significance alone.

## Figures and Tables

**Table 1:** FinnGen R13 infection outcomes used in the proteome-wide MR analysis.

Outcome	Infection-Site Module	Role	Cases	Controls
Pneumonia	Pneumonia/lower respiratory	Primary	84,364	415,822
Bacterial pneumonia	Pneumonia/lower respiratory	Sensitivity	22,740	409,571
Lower-respiratory infection	Pneumonia/lower respiratory	Sensitivity	29,724	470,462
Implicit sepsis	Sepsis/systemic infection	Primary	53,325	430,878
Bacterial sepsis	Sepsis/systemic infection	Sensitivity	22,788	437,107
Other septicemia	Sepsis/systemic infection	Sensitivity	19,048	433,757
Skin/subcutaneous infection	Skin/soft tissue	Primary	31,768	468,418
Cellulitis	Skin/soft tissue	Sensitivity	10,223	468,418
Cutaneous abscess	Skin/soft tissue	Sensitivity	12,480	468,418
Cystitis	Urinary tract	Primary	71,471	392,269
Pyelonephritis	Urinary tract	Sensitivity	35,700	442,323

Note: Abbreviations: MR, Mendelian randomization. Primary outcomes were used for the main site-specific screening; sensitivity outcomes were used to evaluate phenotype coherence within each infection-site module. The sepsis outcome corresponds to the FinnGen implicit sepsis phenotype and should be interpreted as a registry-derived systemic infection phenotype rather than a clinically adjudicated sepsis endpoint.

**Table 2:** Integrated evidence framework for core prioritized protein–infection pairs.

Protein/Evidence Outcome	MR Evidence	Regional Colocalization	Prior Sensitivity	Directionality	Reference-Panel LD	Interpretation
PSCA Cystitis	OR 0.977 (0.967–0.987); *p* = 7.44 × 10^−6^; primary FDR = 0.0169	PP.H4 = 0.954	Stringent PP.H4 = 0.676; robust moderate-to-strong	93.3% supported	MR–top coloc: FIN r^2^ = 0.980; EUR r^2^ = 0.941 (high LD)	Principal novel cystitis-specific, genetically anchored susceptibility candidate. Best viewed as a mechanistic probe rather than an established intervention target; oncology and urogenital safety require evaluation.
UMOD Pyelonephritis; cystitis also supported	Pyelonephritis OR 0.952 (0.934–0.971); *p* = 6.47 × 10^−7^; FDR-all = 0.0030	PP.H4 = 0.995 for pyelonephritis; 0.910 for cystitis	Stringent PP.H4 = 0.949 for pyelonephritis and 0.502 for cystitis; robust	93.1% supported for pyelonephritis; 88.0% for cystitis	MR–top coloc: FIN r^2^ = 0.947; EUR r^2^ = 0.982 (high LD)	Urinary-tract biological anchor supporting renal and urinary host-defense coherence; retained as an anchor rather than the principal novelty claim.
APOE Implicit sepsis; lower-respiratory support	Implicit sepsis OR 0.940 (0.924–0.956); *p* = 9.66 × 10^−13^; primary FDR = 6.59 × 10^−9^	PP.H4 = 0.9998 for implicit sepsis; approximately 1.000 for lower-respiratory infection	Stringent PP.H4 = 0.998 for implicit sepsis; robust strong	94.0% supported for implicit sepsis	MR–top coloc: FIN r^2^ = 0.539, EUR r^2^ = 0.669 (intermediate); UKB-PPP–deCODE: FIN r^2^ = 0.084, EUR r^2^ = 0.060 (low)	Pleiotropy-prone broad host-response or severity-associated locus, not an infection-specific or straightforward therapeutic target. deCODE represents regional pQTL-source follow-up, not proxy-instrument replication.
TNFSF8 Pneumonia; lower-respiratory follow-up	OR 1.142 (1.088–1.199); *p* = 8.54 × 10^−8^; primary FDR = 2.91 × 10^−4^	PP.H4 = 0.544; PP.H3 = 0.456	Stringent PP.H4 = 0.107; prior-sensitive weak	85.4% supported	MR–top coloc: FIN r^2^ = 0.979, EUR r^2^ = 0.992; UKB-PPP–deCODE: FIN r^2^ = 0.957, EUR r^2^ = 0.910 (high LD)	Lower-respiratory follow-up hypothesis. High LD supports proxy-instrument follow-up but does not resolve the moderate, prior-sensitive colocalization architecture; not a near-definitive pneumonia target.

Note: MR evidence is expressed per genetically predicted increase in circulating protein abundance. PP.H4 is the posterior probability of a shared regional causal variant; PP.H3 is the posterior probability of distinct causal variants for the two traits. PP.H4 categories are pragmatic interpretive labels rather than absolute decision boundaries. Reference-panel LD was estimated using the high-coverage 1000 Genomes FIN population as the primary reference and EUR as a sensitivity reference. High LD was defined as r^2^ ≥ 0.80, intermediate LD as 0.20 ≤ r^2^ < 0.80, and low LD as r^2^ < 0.20. Physical proximity alone was not interpreted as evidence of LD or a shared causal variant. TGFB1 and NUDT15 are retained in Supplementary Table S7 as secondary/cautionary signals. FDR, false discovery rate; LD, linkage disequilibrium; MR, Mendelian randomization.

**Table 3:** Site-resolved synthesis of prioritized host-protein signals.

Infection-Site Module	Prioritized Proteins	Evidence Summary	Interpretation
Urinary-tract infection	PSCA	Cystitis-specific MR signal with strong cystitis colocalization; less pyelonephritis support.	Cystitis-specific genetically anchored susceptibility candidate and mechanistic probe; not an established intervention target.
Urinary-tract infection	UMOD	Cystitis and pyelonephritis MR support with strong or robust colocalization.	Urinary anchor supporting renal/urinary host-defense coherence.
Pneumonia/lower-respiratory infection	TNFSF8; APOE	TNFSF8 showed robust pneumonia MR and high-LD proxy-instrument follow-up, but regional colocalization was moderate and prior-sensitive. APOE provided pleiotropy-prone lower-respiratory support.	Lower-respiratory follow-up module with unresolved shared-causal architecture; not definitive therapeutic evidence.
Implicit sepsis/systemic host response	APOE	Strong and robust colocalization for sepsis/lower-respiratory outcomes, but highly pleiotropic.	Pleiotropy-prone broad host-response or severity-associated locus, not an infection-specific or straightforward therapeutic target.
Skin/soft-tissue infection	No convergent lead	No candidate combined FDR-level MR and supportive colocalization.	Negative finding supports the site-specificity of the observed protein architecture.
Supportive/cautionary candidates	TGFB1; NUDT15	TGFB1 tractable but weak/prior-sensitive coloc and broad safety domains; NUDT15 lacks coloc support.	TGFB1 should be interpreted as a tractability-positive but evidence-limited cautionary candidate, whereas NUDT15 is retained as a supplementary MR-only signal.

Note: The table summarizes genetically anchored protein-prioritization evidence and does not establish definitive causal mechanisms or therapeutic effects. The absence of a convergent skin/soft-tissue lead is interpreted as an informative negative finding rather than evidence that circulating proteins are irrelevant to those infections.

## Data Availability

The data analyzed in this study were derived from publicly accessible or publicly available third-party summary-statistic resources. FinnGen R13 infection GWAS summary statistics are available from the FinnGen data release portal. UKB-PPP plasma pQTL summary statistics, deCODE plasma-proteome pQTL summary statistics, ProteoNexus pQTL resources, Open Targets annotations, and UniProt annotations are available through their respective public repositories or data portals. The processed candidate-level results and complete machine-readable output tables supporting this study are provided in the Supplementary Materials and Supplementary Data. Analysis code is available from the corresponding author upon reasonable request.

## Supplementary Materials

The following supporting information can be downloaded at https://www.mdpi.com/article/10.3390/genes17080887/s1, Supplementary tables for infection outcomes, analysis scale, MR signal summaries, colocalization, prior sensitivity, candidate interpretation, safety-domain annotation, QC checks, deCODE pQTL-source follow-up, directionality sensitivity, physical-proximity assessment, and FIN/EUR reference-panel LD analyses. Supplementary Data: Complete machine-readable result tables, including cis-pQTL instruments, harmonized MR outputs, colocalization outputs, prior-sensitivity results, annotation tables, safety-domain results, and candidate-level follow-up results.
