# Supplementary material for: Site-Resolved Plasma-Protein Architecture of Infection Susceptibility: A Cis-pQTL Mendelian Randomization and Colocalization Study
Source: Genes (Basel). 2026 Jul 29;17(8):887. doi: 10.3390/genes17080887 (PMC13512337; doi:10.3390/genes17080887)
Supplement: Supplementary file 1 [file genes-17-00887-s001.zip › Supplementary Material.pdf]

Supplementary Material

Site-resolved plasma protein architecture of infection susceptibility: a cis-pQTL Mendelian randomization and colocalization study

This supplementary document provides formatted supplementary tables supporting the main manuscript. Very large machine-readable tables, including all cis-pQTL instruments, all proteome-wide MR results, SNP-level directionality calculations, regional colocalization outputs, prior-sensitivity analyses, Open Targets/UniProt annotation, and safety-domain results, are provided in Supplementary Data. The supplementary tables support the candidate-prioritization framework that identifies PSCA as the principal cystitis-specific candidate, UMOD as the urinary-tract anchor, APOE as a pleiotropy-prone broad host-response or severity-associated locus, and TNFSF8 as a lower-respiratory follow-up hypothesis.

Supplementary Table S1. FinnGen R13 infection outcome definitions and sample sizes.

Phenocode	Outcome	Site module	Role	FinnGen phenotype	Cases	Controls
J10_PNEUMONIA	Pneumonia	Pneumonia/lower respiratory	Primary	All pneumoniae	84,364	415,822
J10_PNEUMOBACT	Bacterial pneumonia	Pneumonia/lower respiratory	Sensitivity	Bacterial pneumoniae	22,740	409,571
J10_LOWERINF	Lower respiratory infection	Pneumonia/lower respiratory	Sensitivity	Other acute lower respiratory infections	29,724	470,462
AB1_SEPSIS_IMPLICIT_WIDE	Sepsis	Sepsis/systemic infection	Primary	Implicit sepsis	53,325	430,878
AB1_SEPSIS_BACT_IMPL_WIDE	Bacterial sepsis	Sepsis/systemic infection	Sensitivity	Implicit bacterial sepsis	22,788	437,107
AB1_OTHER_SEPSIS	Other septicaemia	Sepsis/systemic infection	Sensitivity	Other septicaemia	19,048	433,757
L12_INFECT_SKIN	Skin/subcutaneous infection	Skin/soft tissue	Primary	Infections of the skin and subcutaneous tissue	31,768	468,418
L12_CELLULITIS	Cellulitis	Skin/soft tissue	Sensitivity	Cellulitis	10,223	468,418
L12_ABSCESS_CUT	Cutaneous abscess	Skin/soft tissue	Sensitivity	Cutaneous abscess, furuncle and carbuncle	12,480	468,418
N14_CYSTITIS	Cystitis	Urinary tract	Primary	Cystitis	71,471	392,269
N14_PYELONEPHR	Pyelonephritis	Urinary tract	Sensitivity	Acute tubulo-interstitial nephritis	35,700	442,323

Note: *Primary outcomes were used for the main site-resolved screen; sensitivity outcomes were used to evaluate phenotype coherence within each infection-site module.*

Supplementary Table S2. Analysis scale, instrument structure, and instrument strength.

Item	Value
Number of FinnGen infection outcomes	11
Number of primary infection outcomes	4
Number of sensitivity infection outcomes	7
Eligible allele-specific cis-pQTL rows after MHC exclusion	1,952
Unique protein–rsID instrument pairs	1,951
Proteins with eligible cis-pQTL instruments	1,950
Proteins with one unique rsID instrument	1,949 (99.95%)
Proteins with two or more unique rsID instruments	1
Median F statistic among unique protein–rsID instruments	941.81 (IQR 250.87–3,529.62)
F-statistic range	45.37–37,472.46
Unique instruments with F < 10	0
Proteins retained after harmonization	1,706
Total harmonized proteome-wide MR tests	18,766
Primary-outcome MR tests	6,824
Candidate proteins taken forward	6
Regional colocalization tests	66
Prior-sensitivity settings	4

Note: *The 1,952 allele-specific rows corresponded to 1,951 unique protein–rsID instrument pairs because the multiallelic GPHA2 variant rs200683903 was represented by two distinct allele-specific records. F-statistic summaries were calculated across unique protein–rsID instruments. MHC, major histocompatibility complex; MR, Mendelian randomization.*

Supplementary Table S3. MR signal summary by infection outcome.

Outcome	Site	Role	Tests	P < 0.001	FDR-all < 0.05	Primary FDR < 0.05	Top protein	Top OR (95% CI)	Top P	Top FDR-all
Pneumonia	Pneumonia/lower respiratory	Primary	1,706	15	1	2	TNFSF8	1.142 (1.088–1.199)	8.54×10^{-8}	5.34×10^{-4}
Bacterial pneumonia	Pneumonia/lower respiratory	Sensitivity	1,706	7	0	0	EBI3_IL27	0.553 (0.419–0.731)	3.10×10^{-5}	0.0645
Lower respiratory infection	Pneumonia/lower respiratory	Sensitivity	1,706	11	1	0	APOE	1.073 (1.050–1.097)	4.06×10^{-10}	3.81×10^{-6}
Implicit sepsis	Sepsis/systemic infection	Primary	1,706	5	1	1	APOE	0.940 (0.924–0.956)	9.66×10^{-13}	1.81×10^{-8}
Bacterial sepsis	Sepsis/systemic infection	Sensitivity	1,706	5	0	0	ARG1	0.832 (0.756–0.916)	1.71×10^{-4}	0.1240
Other septicaemia	Sepsis/systemic infection	Sensitivity	1,706	3	1	0	APOE	1.071 (1.042–1.101)	1.43×10^{-6}	0.0054
Skin/subcutaneous infection	Skin/soft tissue	Primary	1,706	7	0	0	MPI	1.173 (1.083–1.271)	8.70×10^{-5}	0.1025
Cellulitis	Skin/soft tissue	Sensitivity	1,706	3	0	0	PDZD2	0.795 (0.712–0.889)	5.43×10^{-5}	0.0837
Cutaneous abscess	Skin/soft tissue	Sensitivity	1,706	4	0	0	ARHGAP25	0.585 (0.442–0.773)	1.66×10^{-4}	0.1240
Cystitis	Urinary tract	Primary	1,706	13	2	3	PSCA	0.977 (0.967–0.987)	7.44×10^{-6}	0.0233
Pyelonephritis	Urinary tract	Sensitivity	1,706	7	1	0	UMOD	0.952 (0.934–0.971)	6.47×10^{-7}	0.0030

Note: *This table reports the top protein by P value within each outcome. Full proteome-wide MR results are provided in Supplementary Data. FDR, false discovery rate; MR, Mendelian randomization.*

Supplementary Table S4. Primary-outcome FDR-supported MR candidates.

Outcome	Site	Protein	UniProt	cis gene	OR (95% CI)	P	Primary FDR	FDR-all	Mean F
Implicit sepsis	Sepsis/systemic infection	APOE	P02649	APOE	0.940 (0.924–0.956)	9.66×10^{-13}	6.59×10^{-9}	1.81×10^{-8}	14,276.6
Pneumonia	Pneumonia/lower respiratory	TNFSF8	P32971	TNFSF8	1.142 (1.088–1.199)	8.54×10^{-8}	2.91×10^{-4}	5.34×10^{-4}	1,350.8
Cystitis	Urinary tract	PSCA	O43653	PSCA	0.977 (0.967–0.987)	7.44×10^{-6}	0.0169	0.0233	34,471.5
Cystitis	Urinary tract	TGFB1	P01137	TGFB1	0.896 (0.852–0.941)	1.29×10^{-5}	0.0220	0.0346	1,252.7
Pneumonia	Pneumonia/lower respiratory	NUDT15	Q9NV35	NUDT15	1.219 (1.112–1.337)	2.57×10^{-5}	0.0351	0.0602	71.0
Cystitis	Urinary tract	UMOD	P07911	UMOD	0.969 (0.955–0.984)	3.71×10^{-5}	0.0422	0.0697	15,248.2

Note: *Primary FDR was calculated within the four prespecified primary infection outcomes, whereas FDR-all was calculated across all 18,766 harmonized protein–outcome tests. FDR, false discovery rate.*

Supplementary Table S5. Moderate or strong regional colocalization results.

Protein	Outcome	Site	Overlapping SNPs	PP.H4	PP.H3	Coloc level
APOE	lower respiratory infection	pneumonia/lower respiratory	844	1.000	0.000	Strong
APOE	Implicit sepsis	sepsis/systemic infection	844	1.000	0.000	Strong
UMOD	pyelonephritis	urinary tract	831	0.995	0.004	Strong
PSCA	cystitis	urinary tract	822	0.954	0.012	Strong
UMOD	cystitis	urinary tract	830	0.910	0.006	Strong
APOE	other septicaemia	sepsis/systemic infection	844	0.858	0.013	Strong
APOE	pneumonia	pneumonia/lower respiratory	843	0.756	0.003	Moderate
TNFSF8	pneumonia	pneumonia/lower respiratory	1,346	0.544	0.456	Moderate

Supplementary Table S6. Prior-sensitivity classification for manuscript-priority protein–outcome pairs.

Protein	Outcome	Interpretive role	SNPs	Default PP.H4	Conservative PP.H4	Stringent PP.H4	Low p1/p2 PP.H4	Robustness
APOE	Lower respiratory infection	Pleiotropy-prone lower-respiratory support	844	1.000	1.000	1.000	1.000	Robust strong
APOE	Implicit sepsis	Pleiotropy-prone broad host-response/severity signal	844	1.000	1.000	0.998	1.000	Robust strong
UMOD	Pyelonephritis	Upper-UTI supportive urinary anchor	831	0.995	0.989	0.949	0.998	Robust strong
PSCA	Cystitis	Cystitis-specific susceptibility candidate	822	0.954	0.913	0.676	0.965	Robust moderate-to-strong
UMOD	Cystitis	Urinary biological anchor	830	0.910	0.834	0.502	0.915	Robust moderate-to-strong
APOE	Other septicaemia	Systemic host-response sensitivity support	844	0.858	0.752	0.377	0.868	Prior-sensitive moderate
APOE	Pneumonia	Pleiotropy-prone pneumonia support	843	0.756	0.608	0.237	0.758	Prior-sensitive moderate
TNFSF8	Pneumonia	Lower-respiratory follow-up hypothesis	1,346	0.544	0.374	0.107	0.922	Prior-sensitive weak
TGFB1	Cystitis	Tractability-positive cautionary candidate	689	0.237	0.134	0.030	0.251	Prior-sensitive weak
NUDT15	Pneumonia	Supplementary MR-only signal	905	0.001	<0.001	<0.001	<0.001	No robust colocalization

Note: *Stringent PP.H4 refers to the lower shared-causal prior setting. Prior-sensitive signals were interpreted as supportive or hypothesis-generating rather than definitive evidence of a shared causal variant. UTI, urinary-tract infection.*

Supplementary Table S7. Candidate-prioritization summary incorporating reference-panel LD.

Gene	Priority	Evidence outcome	MR OR (95% CI)	PP.H4	Stringent PP.H4	Directionality	MR–top coloc LD	Final interpretation
PSCA	Tier 1 principal candidate	Cystitis	0.977 (0.967–0.987)	0.954	0.676	93.3%	FIN 0.980; EUR 0.941 (high)	Cystitis-specific genetically anchored susceptibility candidate; mechanistic probe with safety caveats.
UMOD	Tier 1 urinary anchor	Pyelonephritis	0.952 (0.934–0.971)	0.995	0.949	93.1%	FIN 0.947; EUR 0.982 (high)	Urinary anchor supporting renal and urinary host-defense coherence.
APOE	Tier 1 cautionary pleiotropic locus	Implicit sepsis	0.940 (0.924–0.956)	1.000	0.998	94.0%	FIN 0.539; EUR 0.669 (intermediate)	Pleiotropy-prone broad host-response or severity-associated locus; not a direct target.
TNFSF8	Tier 2 respiratory follow-up	Pneumonia	1.142 (1.088–1.199)	0.544	0.107	85.4%	FIN 0.979; EUR 0.992 (high)	Lower-respiratory follow-up hypothesis with unresolved shared-causal architecture.
TGFB1	Tier 3 cautionary candidate	Cystitis	0.896 (0.852–0.941)	0.237	0.030	90.9%	FIN 0.049; EUR 0.042 (low)	Tractable but colocalization-, LD- and safety-limited secondary candidate.
NUDT15	Supplementary only	Pneumonia	1.219 (1.112–1.337)	0.001	<0.001	82.7%	FIN 0.053; EUR 0.010 (low)	Supplementary MR-only signal without meaningful regional support.

Note: *The LD column refers to the relationship between the UKB-PPP MR instrument and the top colocalization SNP. FIN was the primary reference population and EUR was used as a sensitivity reference. Candidate roles reflect genetically anchored prioritization and do not imply definitive therapeutic validation.*

Supplementary Table S8. Infection-site interpretation map.

Infection-site module	Protein priority	Evidence interpretation	Interpretive role
Urinary tract infection	PSCA	Cystitis-specific MR, strong colocalization, prior robustness, supportive directionality, and high FIN LD.	Principal cystitis-specific candidate; mechanistic susceptibility probe
Urinary tract infection	UMOD	Coherent cystitis and pyelonephritis associations with strong/prior-robust colocalization, supportive directionality, and high FIN LD.	Urinary biological anchor / coherence module
Systemic infection / implicit sepsis	APOE	Strong MR and regional colocalization but extensive pleiotropy; the deCODE instrument is in low LD with the UKB-PPP instrument.	Cautionary broad host-response / severity-associated locus
Pneumonia / lower respiratory infection	TNFSF8	Strong pneumonia MR and high-LD pQTL-source follow-up, but moderate and prior-sensitive colocalization.	Follow-up hypothesis, not a definitive target
Skin / soft-tissue infection	No convergent lead	No candidate combined FDR-level MR evidence with supportive regional colocalization.	Informative negative module
Supportive / downgraded candidates	TGFB1; NUDT15	TGFB1 has weak colocalization, low LD, and broad safety domains; NUDT15 lacks meaningful colocalization and LD support.	Supplementary / cautionary candidates

Note: *The map separates principal, anchor, follow-up, cautionary, and negative modules. "Follow-up" denotes a hypothesis requiring additional fine-mapping and experimental evaluation rather than a validated therapeutic target.*

Supplementary Table S9. Open Targets PheWAS-like safety summary for prioritized proteins.

Gene	Safety associations	Safety domains	Selected leading safety domains	Selected top safety diseases	Max score
APOE	55	12	Cardiovascular/thrombotic; lipid/metabolic; kidney/urinary; neuropsychiatric; metabolic/endocrine; respiratory; liver/hepatobiliary	Coronary artery disease (0.816); hyperlipoproteinemia type 3 (0.805); lipoprotein glomerulopathy (0.769); Alzheimer disease (0.683); dementia (0.631); familial hypercholesterolemia (0.625); hypercholesterolemia (0.611); type 2 diabetes mellitus (0.592)	0.816
NUDT15	69	17	Cardiovascular/thrombotic; cancer/neoplasm; hematologic; infection; autoimmune/inflammatory; respiratory	Preeclampsia (0.124); brain aneurysm (0.086); cancer (0.084); acute lymphoblastic leukemia (0.083); neoplasm (0.077); Blackfan–Diamond anemia (0.069); severe congenital neutropenia due to CSF3R deficiency (0.060); COVID-19 (0.060)	0.124
PSCA	76	10	Cancer/neoplasm; kidney/urinary; respiratory; hematologic; infection; cardiovascular/thrombotic	Gastric cancer (0.498); gastric carcinoma (0.463); urinary bladder carcinoma (0.349); urinary bladder cancer (0.343); gastric papillary adenocarcinoma (0.319); stomach neoplasm (0.319); ovarian cancer (0.295); breast carcinoma (0.294)	0.498
TGFB1	76	17	Autoimmune/inflammatory; cancer/neoplasm; hematologic; kidney/urinary; cardiovascular/thrombotic; respiratory; infection	Inflammatory bowel disease, immunodeficiency, and encephalopathy (0.709); myelodysplastic syndrome (0.579); cancer (0.533); anemia (0.532); beta-thalassemia (0.486); coronary artery disease (0.464); IL10-related early-onset inflammatory bowel disease (0.453); cystic fibrosis (0.444)	0.709
TNFSF8	64	18	Autoimmune/inflammatory; respiratory; kidney/urinary; cancer/neoplasm; infection; liver/hepatobiliary; hematologic; cardiovascular/thrombotic	Crohn disease (0.380); asthma (0.351); IgA glomerulonephritis (0.341); respiratory system disease (0.324); vulvar benign neoplasm (0.305); pneumonia (0.222); inflammatory bowel disease (0.189); autoimmune disease (0.165)	0.380

Gene	Safety associations	Safety domains	Selected leading safety domains	Selected top safety diseases	Max score
UMOD	75	13	Kidney/urinary; cardiovascular/thrombotic; hematologic; metabolic/endocrine; infection; autoimmune/inflammatory	Familial juvenile hyperuricemic nephropathy type 1 (0.845); kidney disease (0.714); autosomal dominant medullary cystic kidney disease (0.672); chronic kidney disease (0.642); kidney failure (0.630); essential hypertension (0.593); nephrolithiasis (0.568); urolithiasis (0.508)	0.845

Note: *The safety scan is a knowledgebase/PheWAS-like prioritization layer, not individual-level safety validation. To improve readability, selected leading domains and diseases are shown here; the complete association lists and scores are provided in Supplementary Data.*

Supplementary Table S10. Interpretive safeguards for prioritized candidates.

Issue	Interpretation safeguard
ProteoNexus pQTL versus UKB-PPP instruments	ProteoNexus pQTL lead variants were not always identical to the UKB-PPP cis-pQTL instruments; colocalization was therefore interpreted as regional shared-signal evidence rather than exact-instrument validation.
APOE pleiotropy	APOE was interpreted as a pleiotropy-prone broad host-response or severity-associated locus rather than an infection-specific or straightforward therapeutic target.
TGFB1 tractability and safety	TGFB1 had favorable tractability annotation but weak/prior-sensitive colocalization, low local LD, and broad immune, fibrotic, oncologic, cardiovascular, and respiratory safety concerns.
TNFSF8 prior sensitivity	TNFSF8 was interpreted as a lower-respiratory follow-up hypothesis because colocalization was moderate and prior-sensitive despite robust MR and high-LD proxy-instrument follow-up.
Skin/soft-tissue null finding	The absence of a convergent skin/soft-tissue lead was treated as an informative negative module rather than a failure of analysis.
Knowledgebase safety scan	Open Targets safety-domain results were used as a knowledgebase/PheWAS-like prioritization layer and were not considered individual-level safety validation.

Note: *These safeguards guide interpretation of the prioritized candidates and are not additional statistical significance thresholds.*

Supplementary Table S11. Candidate-level deCODE pQTL-source follow-up with LD relationships to the UKB-PPP instruments.

Gene	deCODE SNP	UKB-PPP SNP	FIN r²	EUR r²	deCODE F	FinnGen outcome	OR (95% CI)	P value	Interpretation
APOE	rs71352239	rs429358	0.084	0.060	778.9	Other septicaemia	1.032 (0.941–1.132)	0.50623	Regional pQTL-source follow-up; low LD with the UKB-PPP instrument, not proxy-instrument replication.
APOE	rs71352239	rs429358	0.084	0.060	778.9	Implicit sepsis	0.946 (0.893–1.003)	0.06222	Borderline same-direction regional follow-up; low LD precludes direct instrument replication.
APOE	rs71352239	rs429358	0.084	0.060	778.9	Lower respiratory infection	1.065 (0.989–1.146)	0.09787	Regional, nonuniform pQTL-source follow-up.
APOE	rs71352239	rs429358	0.084	0.060	778.9	Pneumonia	1.018 (0.970–1.068)	0.46415	Regional, nonuniform pQTL-source follow-up.
TNFSF8	rs1006026	rs10081728	0.957	0.910	280.8	Lower respiratory infection	1.138 (1.006–1.287)	0.03924	High-LD proxy-instrument follow-up; does not resolve prior-sensitive colocalization.
TNFSF8	rs1006026	rs10081728	0.957	0.910	280.8	Bacterial pneumonia	1.243 (1.078–1.432)	0.00268	High-LD proxy-instrument follow-up; does not resolve prior-sensitive colocalization.
TNFSF8	rs1006026	rs10081728	0.957	0.910	280.8	Pneumonia	1.229 (1.135–1.331)	$< 1 \times 10^{-4}$	High-LD proxy-instrument follow-up; supports follow-up status, not definitive target designation.

Note: *Candidate-level follow-up used genome-wide significant deCODE plasma-proteome cis-pQTL instruments. APOE showed partial/borderline sepsis-direction consistency with nonuniform lower-respiratory estimates, whereas TNFSF8 showed concordant lower-respiratory support. FIN was the primary reference population and EUR was used as a sensitivity reference.*

Supplementary Table S12. Directionality sensitivity, physical proximity, and reference-panel LD for prioritized protein–outcome pairs.

Gene	Outcome	Interpretive role	Directionality	MR SNP	Top coloc SNP	Distance (kb)	PP.H4	Stringent PP.H4	FIN r²	EUR r²	Interpretation
APOE	Other septicaemia	Systemic host-response sensitivity support	92.4%	rs429358	rs4420638	11.0	0.858	0.377	0.539	0.669	Intermediate LD; regional relationship, not interchangeable instruments.
APOE	Implicit sepsis	Pleiotropy-prone broad host-response/severity signal	94.0%	rs429358	rs4420638	11.0	1.000	0.998	0.539	0.669	Robust colocalization with intermediate LD; interpret cautiously because of pleiotropy.
APOE	Lower respiratory infection	Pleiotropy-prone lower-respiratory support	92.5%	rs429358	rs4420638	11.0	1.000	1.000	0.539	0.669	Strong regional evidence with intermediate LD; broad host-response interpretation.
APOE	Pneumonia	Pleiotropy-prone pneumonia support	93.2%	rs429358	rs4420638	11.0	0.756	0.237	0.539	0.669	Prior-sensitive regional signal; not infection-specific.
NUDT15	Pneumonia	Supplementary MR-only signal	82.7%	rs12429926	rs12427929	179.2	0.001	<0.001	0.053	0.010	Low LD and no meaningful colocalization; supplementary MR-only signal.
PSCA	Cystitis	Cystitis-specific susceptibility candidate	93.3%	rs2976388	rs1045574	3.7	0.954	0.676	0.980	0.941	High LD strengthens local coherence but does not establish mechanism or therapeutic effect.

Gene	Outcome	Interpretive role	Directionality	MR SNP	Top coloc SNP	Distance (kb)	PP.H4	Stringent PP.H4	FIN r²	EUR r²	Interpretation
TGFB1	Cystitis	Tractability-positive cautionary candidate	90.9%	rs73045269	rs1800469	35.1	0.237	0.030	0.049	0.042	Low LD confirms that physical proximity alone is not shared-signal evidence.
TNFSF8	Pneumonia	Lower-respiratory follow-up hypothesis	85.4%	rs10081728	rs726657	0.7	0.544	0.107	0.979	0.992	High LD but moderate and prior-sensitive colocalization; requires fine-mapping.
UMOD	Cystitis	Urinary biological anchor	88.0%	rs12917707	rs13335818	7.9	0.910	0.502	0.947	0.982	High LD and strong local coherence.
UMOD	Pyelonephritis	Upper-UTI supportive urinary anchor	93.1%	rs12917707	rs13335818	7.9	0.995	0.949	0.947	0.982	High LD and robust strong colocalization.

Note: *Directionality was assessed as a Steiger-like sensitivity analysis using the validated merged pQTL–FinnGen regional SNP set. It supports but does not prove the protein-to-infection direction. Distance is the physical distance between the UKB-PPP MR SNP and the ProteoNexus/FinnGen top colocalization SNP. FIN was the primary reference population and EUR was used as a sensitivity reference. LD, linkage disequilibrium; MR, Mendelian randomization; UTI, urinary-tract infection.*
